# Antipsychiatry: Meeting the challenge

**DOI:** 10.4103/0019-5545.43048

**Published:** 2005

**Authors:** Nimesh G. Desai

The healthy nature of any debate with ‘proponents for’ and ‘opponents against’ a theme or a subject, in any form, and its beneficial effects are well known and recognized. The value of such debates on issues having importance in the growth of any discipline of science, including those of the medical sciences is immense, and yet it would be unthinkable to be dealing with ‘anti-cardiologists’ or ‘anti-paediatricians’! The fact that ‘antipsychiatry’ has existed in one form or the other for some time, and indeed has sometimes been vehement enough to approach psychiatry as a demon to be exorcised, is noteworthy.

The opposition to the involuntary custodial care in psychiatric hospitals, which has been one of the major reservations from the antipsychiatry groups, is quite understandable, as is the demand for a more humane approach to psychiatry; but it has often gone beyond these basic issues. The range of opinions and strong advocacy positions within the loosely formed group of antipsychiatrists, and the impact it has had on psychiatry requires to be understood, besides recognizing the enormity of the challenge and the need to meet the challenge effectively and levelly.

Although there were criticisms of the theory and practice of psychiatry in the nineteenth century, the strident criticisms from different perspectives articulated during the second half of the twentieth century have been collectively termed as ‘antipsychiatry’ by David Cooper, a South African psychoanalyst.^1^,^2^ The first of the major steps in this direction was taken by a science fiction writer, L. Ron Hubbard, who founded the Church of Scientology in 1950, with the goal of ‘eradicating psychiatry from the face of this earth’.^3^ His book, *Dianetics: The modern science of mental health*, prescribed the techniques of rundown (a series of steps to produce a certain end result), introspection rundown designed to handle a psychotic break (by isolating the person wholly with all the attendants completely muzzled, till the person is out of his psychotic break) and auditing (looking back through a person's past to find some memory that is causing problems to the person at present).

In 1969, the Citizens Commission of Human Rights (CCHR) was founded by scientology ‘to expose the evils of psychiatry’.^4^ The attacks on psychiatry and psychiatrists by scientology and the CCHR have continued, and indeed have increased with many celebrities joining hands. One striking example is of the Hollywood actor Tom Cruise who advocated in January 2004: ‘I think psychiatry should be outlawed.’ On the other hand, the undesirable impact of following scientology has also been noticed, a striking case being of one Lisa McPherson who died in 1995, brought dead to the hospital with significant weight loss, bruises and bug bites, having been put on ‘introspection rundown’ to handle a psychotic break.^5^

The second major trend in antipsychiatry has been of psychiatrists and sociologists who have, for different reasons, questioned not only the coercive authority of psychiatrists and psychiatry in diagnosing and ‘putting people away’ for treatment in hospitals, but also the very basis of psychiatric diagnosis and the increasing ‘medicalization’ of mental illnesses. Interestingly, the ‘seige from within’ came almost simultaneously from two psychoanalysts across the Atlantic Ocean. In the UK, R.D. Laing, who started his writings in 1960, saw mental disorders or at least schizophrenia as an understandable and even normal response of sensitive individuals to a ‘mad’ world.^6^–^9^ He emphasized the importance of freedom and subjectivity over determinism, and believed that cure would occur when patients felt that they were free to make choices. For the diagnostic processes of modern psychiatrists, he used the term ‘psychiatrosis’ as a new type of mental disorder. About the same time, in the USA, Thomas Szasz in his writings questioned the existence of mental illness and medical diseases since they did not satisfy the Koch's postulates for tuberculosis and other infectious diseases. As a defender of radical individualism, he opposed involuntary hospitalization and treatment and argued that ‘whereas in modern medicine new diseases were discovered, in modern psychiatry they are invented’ and that mental disorders were no more than ‘myths’ of fraudulent impositions perpetuated by psychiatrists whose central intention was to preserve their privileged professional status.^10^–^13^

The dissent from sociological thought and perspective came from two sociologists in the USA, viz. Erving Goffman and Thomas Scheff. In his book *Asylums*, Goffman described what he saw at St Elizabeth's, an institution with over 6000 patients with psychiatric illnessess.^14^ He opined that psychiatrists used asylums as brainwashing machines to control disturbing individuals. His observations did draw attention to some serious weaknesses in mental hospitals, leading to desirable reforms, but unfortunately he overstated the point to the extent that there was no such thing as mental illness. The experience with deinstitutionalization in Italy and other countries has not yielded the benefits of exaggerated emphasis against all kinds of institutions, and has established the need and relevance for the process of reform of mental hospitals to become mental health institutions serving key functions. The process of deinstitutionalization, when carried too far, has had its undesirable effect in causing a rapid growth in the number of mentally ill homeless individuals.^15^ In Thomas Sheff's ‘labelling theory’, individuals are ‘labelled as deviant or mentally ill because they have isolated social norms or their behaviour is what a society considers unacceptable behaviour’. It was argued by Sheff that ‘most chronic mental illness is, at least in part, a social role’.^16^

The third major force in antipsychiatry started in England in the early 1970s with the formation of the Mental Patients Union, led by the so-called ‘survivors’ of psychiatry, which has also gained its geographical and ideological influence, with the World Network of Users and Survivors of Psychiatry (WNUSP) being at the forefront of the movement.^17^ Although their activities often deny the reality of mental illness and criticize the lack of sensitivity on the part of psychiatrists, the movement has led to bridging the gap between psychiatrists and users of their services in some parts of the world,^18^ and can further help the process globally. This group has also recently been joined by human rights activists including lawyers, who are primarily opposed to any active or passive violation of human rights including involuntary hospitalization and treatment.

As is evident, the different forces in antipsychiatry movement, even if they are not based on common theory or conceptualization, have been opposed to the power wielded by psychiatry and psychiatrists over peoples' lives, the lack of a humane approach and undue medicalization of psychiatry, and advocating the need for respecting and actively promoting some basic rights of mentally ill persons. It should be readily accepted that these are concepts we cannot disagree with or shy away from! Some of the practical implications of these concepts, e.g. the right to refuse hospitalization or treatment, may bring forth issues requiring negotiation, and some other tenets of the antipsychiatry school of thought, viz. demonizing psychiatry or challenging the very basis of diagnosis and treatment, may be more difficult to deal with, not only in our own limited professional interest, but genuinely in the interest of persons with mental illnesses and their families. The fundamental concept of ‘respect for the person’, from where the antipsychiatry thoughts and arguments come, has unfortunately been, in general, alien to psychiatry and psychiatrists, barring exceptions. The inherent, erstwhile paternalism of medicine does seem to have become so accentuated in psychiatry as to lead to nonconsensual approach in treatment being the norm. Some of the negative experiences of users of psychiatry and the ringside observers leading to various ideologies of antipsychiatry and some of the dangers portrayed are too real to be ignored! The issues involved in involuntary hospitalization and treatment, not to mention the multifarious misuse and abuse of the related provisions, have to be recognized with candour. Szasz's description of modern psychiatry ‘inventing’ diseases does not seem too far-fetched in view of the ever-expanding, seamless boundaries of the current systems of classification!

The benefits of the larger conceptual and ideological opposition of antipsychiatry to the practice of psychiatry at the macro level have been remarkable in the development and refinement of psychiatry.^2^,^18^ To be a believer and a practitioner of multidisciplinary mental health, it is not necessary to reject the medical model as one of the basics of psychiatry. Sometimes neither the psychiatrists nor the antipsychiatrists realize that the medical model of psychiatry, and the claim that it is a neuroscience, can be synchronous with the larger concepts of multidisciplinary mental health care and participatory mental health care. Some of the challenges and dangers to psychiatry are not so much from the avowed antipsychiatrists, but from the misplaced and misguided individuals and groups in related fields. The implicit danger and challenge at micro levels of mental health teams or centres are possibly more difficult to deal with as compared to the macro-level ideological challenge.

It has been argued that after the 1970s, the antipsychiatry movement became increasingly less influential, due in particular to the advances in psychiatry and neurobiology and in general to the improvements in the efficacy of available treatment.^19^ Yet, in some countries it still remains influential in formulation of mental health policy. There is a fair possibility of the disparate forces of antipsychiatry gaining momentum and influence in countries of the developing world such as India, not in the least contributed to by the effects of globalization. Such a development could be hazardous for the society in general, given the possibility that the debates often do not remain ideological but tend to get acrimonious and personalized easily, and the benefits of the science of psychiatry have not been far reaching as yet for other advocacy groups such as families of treated persons to get their voice heard equally. On the other hand, the fact that the understanding and acceptance of psychiatry is steadily improving and that some good work in community participation in mental health services has already begun may be the encouraging factors in meeting the challenge of antipsychiatry. The nature and enormity of the challenge is evident, and yet it needs to be met with as levelly and meaningfully as possible. The response pattern of nonchalance or acrimonious name-calling or labelling is not likely to help, nor can the psychiatrists permit themselves to feel demoralized or threatened. In addition to competence and evidence base for the practice of psychiatry, psychiatrists and their allies—the patients and their families who avail of psychiatry services do not see themselves as ‘survivors’ but ‘beneficiaries’—could do well as to understand the criticisms emanating from these antipsychiatry groups, and the processes contributing to such criticisms. As it so happens, there are millions who have benefited from services provided by psychiatrists, even if they remain silent, or even unaware, bystanders of this discourse on psychiatry and antipsychiatry. At the same time, the need to listen attentively to the voices of dissatisfaction or dissent, however small, is paramount, especially if the discourse is dispassionate and with mutual respect. Moreover, the willingness to accept the valid criticisms and the possible abuses or ‘wrongs’ of psychiatry, as well as to be open to a dialogue or multilogue and review of practices if required would not only be strategically sound but also ethically warranted. Many ideas and observations from antipsychiatry are worthy of careful consideration, even if overstated or exaggerated. It may not be easy for psychiatrists, but the time is bygone when psychiatrists could abrogate to themselves the right to know and decide what is best for mentally ill persons. The need is not to react defensively or in counter-offence, but ‘to democratize mental health by linking progressive service development to a debate about contests, values and partnerships’, as Bracken and Thomas put forth in their view of ‘postpsychiatry’, which takes the debate beyond the conflict between psychiatry and antipsychiatry, distancing itself from the therapeutic implications of antipsychiatry.^20^ Let us recognize and meet the challenge, so as to accommodate all possible viewpoints, and enrich the concept and practice of psychiatry for human welfare.
